# Genome-Wide Identification of B-Box Family Genes and Their Potential Roles in Seed Development under Shading Conditions in Rapeseed

**DOI:** 10.3390/plants13162226

**Published:** 2024-08-11

**Authors:** Si Chen, Yushan Qiu, Yannong Lin, Songling Zou, Hailing Wang, Huiyan Zhao, Shulin Shen, Qinghui Wang, Qiqi Wang, Hai Du, Jiana Li, Cunmin Qu

**Affiliations:** 1 Integrative Science Center of Germplasm Creation in Western China (CHONGQING) Science City, College of Agronomy and Biotechnology, Southwest University, Chongqing 400715, China; cs19960301@email.swu.edu.cn (S.C.); qiuyushan2003@163.com (Y.Q.); 15960619716@163.com (Y.L.); zousonglin0618@163.com (S.Z.); w1299453627@email.swu.edu.cn (H.W.); zhaohuiyan@swu.edu.cn (H.Z.); ssl7159@email.swu.edu.cn (S.S.); cherylw@email.swu.edu.cn (Q.W.); 13228682895@163.com (Q.W.); haidu81@126.com (H.D.); 2 Academy of Agricultural Sciences, Southwest University, Chongqing 400715, China; 3 Engineering Research Center of South Upland Agriculture, Ministry of Education, Chongqing 400715, China

**Keywords:** B-box (BBX) family genes, triangle of U, *Brassica napus* L., light response, seed coat color

## Abstract

B-box (BBX) proteins, a subfamily of zinc-finger transcription factors, are involved in various environmental signaling pathways. In this study, we conducted a comprehensive analysis of BBX family members in *Brassica* crops. The 482 BBX proteins were divided into five groups based on gene structure, conserved domains, and phylogenetic analysis. An analysis of nonsynonymous substitutions and (Ka)/synonymous substitutions (Ks) revealed that most BBX genes have undergone purifying selection during evolution. An analysis of transcriptome data from rapeseed (*Brassica* napus) organs suggested that BnaBBX3d might be involved in the development of floral tissue-specific RNA-seq expression. We identified numerous light-responsive elements in the promoter regions of BnaBBX genes, which were suggestive of participation in light signaling pathways. Transcriptomic analysis under shade treatment revealed 77 BnaBBX genes with significant changes in expression before and after shading treatment. Of these, BnaBBX22e showed distinct expression patterns in yellow- vs. black-seeded materials in response to shading. UPLC-HESI-MS/MS analysis revealed that shading influences the accumulation of 54 metabolites, with light response BnaBBX22f expression correlating with the accumulation of the flavonoid metabolites M46 and M51. Additionally, BnaBBX22e and BnaBBX22f interact with BnaA10.HY5. These results suggest that BnaBBXs might function in light-induced pigment accumulation. Overall, our findings elucidate the characteristics of BBX proteins in six *Brassica* species and reveal a possible connection between light and seed coat color, laying the foundation for further exploring the roles of BnaBBX genes in seed development.

## 1. Introduction

The B-box (BBX) gene family, a subfamily of zinc-finger transcription factor genes, is the most important and largest gene family in plants [1]. BBX transcription factors play major roles in seedling photomorphogenesis, plant development, and both abiotic and biotic stress tolerance [1,2]. BBX domains in plants can be grouped into two types according to the spacing between the zinc-binding residues and the consensus sequence: B-box1 (C-X2-C-X_7-8_-C-X_2_-D-X-A-X-L-C-X_2_-C-D-X_3_-H) and B-box2 (C-X_2_-C-X_3_-P-X_4_-C-X_2_-D-X_3_-L-C-X_2_-C-D-X_3_-H). The B-box domain functions in protein–protein interactions and transcriptional regulation during plant signaling. BBX proteins also possess a CCT (CONSTANS [CO], CO-like, TIMING OF CAB1 [TOC1]) domain at their C-termini [1,3].

In *Arabidopsis* (*Arabidopsis thaliana*), the BBX family comprises 32 members, classified into five groups based on the presence of B-box and CCT domains [4,5]: Groups Ⅰ (AtBBX1–AtBBX6) and Ⅱ (AtBBX7–AtBBX13) members contain two distinct B-box domains and a CCT domain at their C-termini; Group III (AtBBX14–AtBBX17) proteins comprise only one B-box and one CCT domain; Group Ⅳ (AtBBX18–AtBBX25) members just have two B-box domains; and Group Ⅴ proteins (AtBBX26–AtBBX32) possess one single B-box domain [4]. The transcript abundance and activity of CO/BBX1, the first plant BBX protein identified in Arabidopsis, change in response to light [6,7]. BBX proteins might regulate flowering by directly and indirectly regulating the expression of *FLOWERING LOCUS T* (*FT*) in species including Arabidopsis [8], rice (*Oryza sativa*) [9], barley (*Hordeum vulgare*) [10], potato (*Solanum tuberosum*) [11], *Chrysanthemum morifolium* [12], pineapple (*Ananas comosus* (L.) Merr.) [13], and sugar beet (*Beta vulgaris*) [14].

In addition, increasing evidence indicates that BBX proteins play roles in plant responses to various environmental signals, such as light-responsive flavonoid accumulation. For example, COL1/AtBBX2 and COL2/AtBBX3 have little effect on flowering time but influence light input pathway(s) in *Arabidopsis* [15]; AtBBX31 and AtBBX32 function as negative regulators of light responses [16,17]; and AtBBX20-AtBBX25 and AtBBX28 interact with HYPOCOTYL 5 (HY5) to regulate photomorphogenesis [18,19,20,21,22]. Most BBX family members also participate in plant responses to abiotic stress, with evidence for roles in salt-stress resistance in *Arabidopsis* [23,24], salt and drought stress in peanut (*Arachis hypogaea* L.) [25], and drought stress tolerance related to the abscisic acid response in *Chrysanthemum* [26,27]. Collectively, these observations reveal that BBX proteins have widespread functions in orchestrating plant growth and development, as well as indispensable roles in plant responses to both biological and non-biological stress factors.

Besides affecting plant morphogenesis, light is a significant external factor influencing flavonoid accumulation, and BBX family proteins are key regulators in anthocyanin biosynthesis in *Arabidopsis*, apple, pear, and strawberry [18,28,29,30,31,32,33,34,35,36]. MdCOL4 suppresses the expression of genes encoding key enzymes involved in anthocyanin biosynthesis, thereby inhibiting its accumulation in apple under UV-B [37]. PpBBX18 and PpBBX21 antagonistically regulate anthocyanin biosynthesis in pear peel under light treatment [38].

We previously used UPLC-HESI-MS/MS to identify 35 phenolic compounds in flavonoid extracts, finding that these compounds might be associated with seed coat coloration [39]. Light-colored seed coats, a trait associated with higher seed oil yield and quality, are important for the *Brassica* oilseed industry [40,41]. Rapeseed (*Brassica napus* L., AACC, 2n = 38) is a significant oilseed plant for human consumption and feed in China and other countries [42,43]. Seed coat color in *B. napus* is a quantitative trait that is highly susceptible to environmental factors, but its underlying molecular mechanisms remain unclear [44]. However, to date, little is known about the members of the BBX family in *Brassica* U-triangle species or how these proteins affect seed coat color.

In this study, we identified BBX gene family members from six *Brassica* U-triangle species (the diploid *Brassica rapa*, Bra, genotype AA; *Brassica nigra*, Bni, genotype BB; *Brassica oleracea*, Bol, genotype CC; and the tetraploid *Brassica juncea*, Bju, genotype AABB; *Brassica napus*, Bna, genotype AACC; and *Brassica carinata*, Bca, genotype BBCC) and analyzed their evolutionary relationships, gene structures, conserved domains, and promoter cis-elements. In addition, we performed spatiotemporal expression analysis and cis-element analysis among BnaBBX family genes. Our findings provide a theoretical foundation for future comprehensive studies of the functional differentiation of the BBX gene family in *Brassicaceae* plants during evolution. Our study also provides insights into the mechanisms underlying the relationship between seed coat color and light via yeast two-hybrid assays, UPLC-HESI-MS/MS analysis, and qRT-PCR analysis in *B. napus*.

## 2. Results

### 2.1. Identification of BBX Family Genes in Brassica U-Triangle Species

To identify putative BBX proteins in the six *Brassica* species comprising the triangle of U, *B. rapa*, *B. nigra*, *B. oleracea*, *B. juncea*, *B. napus*, and *B. carinata*, we performed a BLASTp search of data from these species using the 32 *Arabidopsis* BBX protein sequences obtained from the TAIR database as queries. After analyzing the conserved domains using NCBI-CDD, HMMER, and InterPro, we utilized BRAD-Syntenic Gene @ subgenomes and the Quick Find Best Homology module in TBtools to further confirm the BBX protein sequences in these species. Using these approaches, we identified novel members of the BBX family that possess partial structural domains typical of the family, such as the CCT domain. Ultimately, we identified 52 BraBBX, 56 BniBBX, 56 BolBBX, 109 BjuBBX, 113 BnaBBX, and 96 BcaBBX genes and named these BBX genes based on their closest homologs in Arabidopsis. The *Brassica* BBX family proteins range from 64 to 875 amino acids (aa) long, with a molecular weight ranging from 7 to 97.83 kDa (Table S1). Most Brassica BBX proteins have a theoretical isoelectric point (pI) value of less than 7. BniBBX28c possesses the lowest PI value (4.13), while BnaBBX6e has the highest PI value (10.97) (Table S1). Most *Brassica* U-triangle BBX proteins have an instability index greater than 40, indicating that they are unstable. All BBX proteins of these six *Brassica* species are hydrophilic, with a grand average of hydropathicity (GRAVY) values of less than 0. We also predicted the in-silico subcellular localizations of all 514 BBX proteins and determined that they all localize to the nucleus (Table S1).

### 2.2. Phylogenetic Relationships of BBXs in Arabidopsis and the Brassica U-Triangle Species

**Figure 1:**
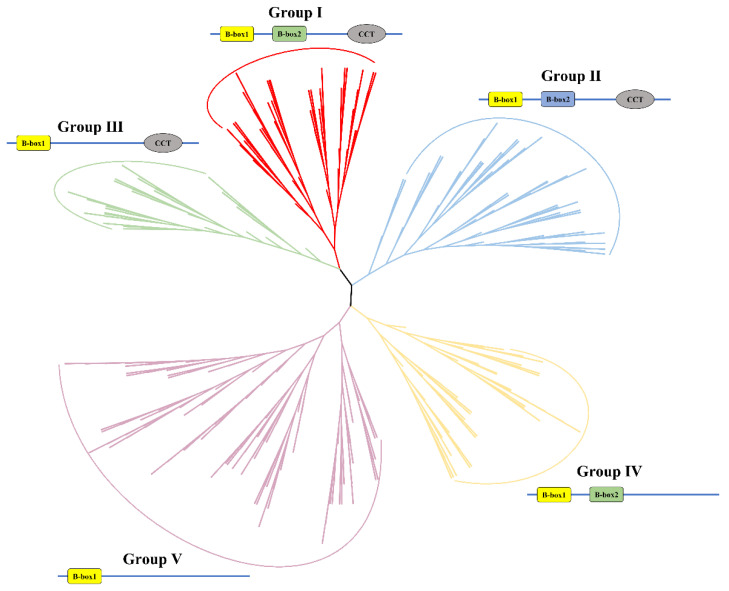
Phylogenetic tree of B-box genes from *Arabidopsis* and six *Brassica* U-triangle species. The phylogenetic tree, constructed using the protein matrix with iq-tree, is grouped into five clades (I–V) labeled with different colors. The phylogenetic tree was visualized using B-box genes from Arabidopsis, *B. rapa*, *B. nigra*, *B. oleracea*, *B. juncea*, *B. napus*, and *B. carinata*.

To elucidate the phylogenetic relationship and divergence of the BBX family, we constructed a maximum-likelihood (ML) tree using iq-tree. The 482 *Brassica* BBXs were grouped into the five different clades (Group I–V) together with their homologs in Arabidopsis, as previously described (Figure 1). We also constructed an ML tree using protein sequences from the 164 *Brassica* BBXs encoded in the A subgenome, 153 *Brassica* BBXs encoded in the B subgenome, and 165 *Brassica* BBXs encoded in the C subgenome in the six *Brassica* U-triangle species. The BBX proteins encoded in all subgenomes were also divided into five groups (Figures S1–S3). Each subgenome contains a similar number of BBX genes, which vary from Group I to V (Figure S4). The B genome possesses the fewest BBX genes, and Groups Ⅱ and IV contain the most.

### 2.3. Conserved Motifs, Conserved Domains, and Gene Structures of BBX Family Members in Brassica U-Triangle Species

To better understand the functions, diversity, and conservation of the 482 *Brassica* BBX proteins, we divided these proteins into three groups (A, B, and C subgenomes) based on the chromosomal locations of their corresponding genes. Using the online MEME suite, we identified 10 conserved motifs, which we named motif 1 to motif 10 based on their order in the proteins (Figure S5). Most BBX proteins encoded in both the A and B subgenomes possess five motifs (Figures S6B and S7B). However, the majority of BBX members from the C subgenome contain four motifs (Figure S8B). In addition, we detected a large difference in the sizes of conserved motifs among proteins encoded in the A, B, and C subgenomes. Motif 7 is the largest motif (50 aa long) of the A-subgenome group, while motifs 1, 6, 8, and 9 are the largest motifs (50 aa each) in the B-subgenome group (Figure S5A,B). The largest conserved motif in the C subgenome is only 41 aa long (Figure S5C). These differences suggest that different biological functions might exist within the A, B, and C subgenomes.

**Figure 2:**
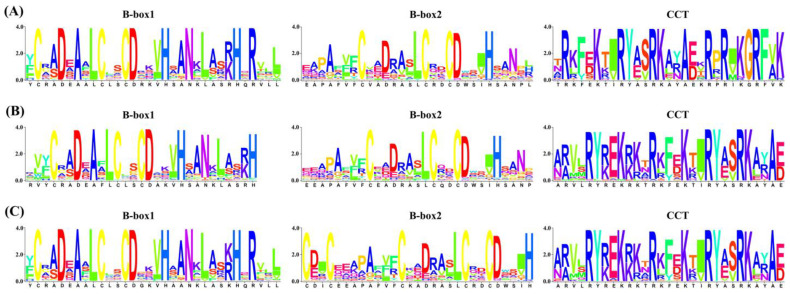
WebLogos of conserved domains of BBX family members in six *Brassica* plants. (**A**–**C**) Typical WebLogos of B-box1, B-box2, and CCT domains in the A, B, and C subgenomes. The *x*-axis depicts the conserved sequences of the structural domains, where the height of each letter signifies the degree of conservation of each residue across all proteins. The *y*-axis indicates the relative entropy scale, which represents the conservation degree of each amino acid.

An investigation of the conserved domains of the *Brassica* BBX family indicated that some proteins retained only a portion of their structural domains compared to other proteins in the same group. For example, some BBX proteins in Group I and Group III lack one B-box domain (Figure S6C). In the B-subgenome group, 7 BBX proteins contain one less B-box domain than others in the same group, and 1 BBX protein lacks a CCT domain (Figure S7C). Two BBX proteins (BolBBX25a and BolBBX26b) in Group IV and three BBX13 proteins encoded in the C subgenome lack one B-box domain (Figure S8C). These results reveal the diversity and intricate history of genome evolution. Furthermore, the B-box domains and CCT domain share high sequence similarity among all subgenomes (Figure 2), demonstrating the conservation of biological functions throughout evolution.

Analyzing the differences in gene structure is essential for studying the evolution of multigene families. We performed a structural analysis of *Brassica* BBX genes based on their coding sequences. The *Brassica* BBX genes on the same branches of the phylogenetic trees contain equal numbers of exons and introns. Members of the A subgenome contain anywhere from 1 to 15 exons (Figure S6C), members of the B subgenome contain 1–12 exons, and members of the C subgenome contain 1–7 exons (Figures S7C and S8C). These findings underline the differentiated biological functions of BBX genes across the A, B, and C subgenomes.

### 2.4. BBX Genes in Brassica U-Triangle Species Experience Purifying Selection

**Figure 3:**
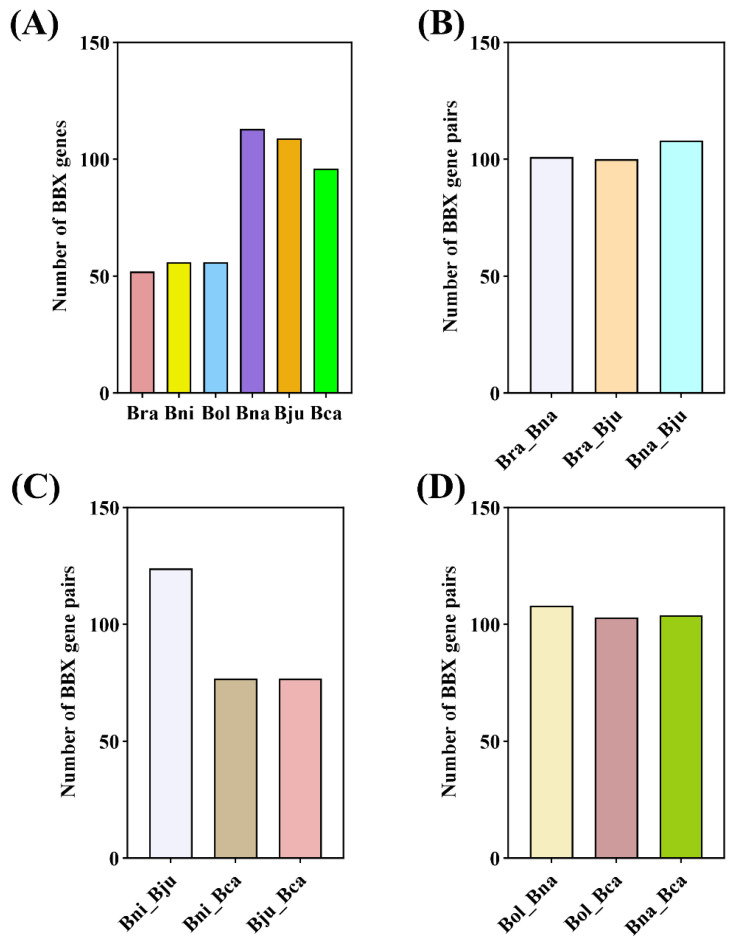
Number of BBX family gene pairs in the six *Brassicaceae* species. (**A**) Number of BBX genes in *B. rapa* (Bra), *B. nigra* (Bni), *B. oleracea* (Bol), *B. napus* (Bna), *B. juncea* (Bju), and *B. carinata* (Bca). (**B**) Number of BBX family gene pairs in the A subgenome. (**C**) Number of BBX family gene pairs in the B subgenome. (**D**) Number of BBX family gene pairs in the C subgenome.

We analyzed the synteny relationships among the *Brassica* BBX family genes in the A, B, and C subgenomes (Figure 3A). Orthologous genes in subgenome A are found between *B. rapa* and *B. napus* (101 pairs of orthologous genes), *B. rapa* and *B. juncea* (100 pairs), and *B. juncea* and *B. napus* (108 pairs) (Figure 3B). Those in subgenome B are found in *B. nigra* and *B. juncea* (124 pairs), *B. nigra* and *B. carinata* (77 pairs), and *B. juncea* and *B. carinata* (77 pairs) (Figure 3C). Those in subgenome C are from *B. oleracea* and *B. napus* (108 pairs), *B. oleracea* and *B. carinata* (103 pairs), and *B. napus* and *B. carinata* (104 pairs) (Figure 3D). BBX genes tend to be concentrated in regions with higher gene density (Figures S9–S11). Additionally, we calculated the ratios of nonsynonymous substitutions (Ka) and synonymous substitutions (Ks) for the orthologous BBX gene pairs to determine the evolutionary constraints affecting the BBX gene pairs. Most Ka/Ks ratios ranged from 0.1 to 0.3, suggesting that BBX family genes in *Brassica* U-triangle species might have undergone strong purifying selection following the duplication events that gave rise to the tetraploid species of the U triangle (Tables S2–S4).

### 2.5. Expression Patterns of BnaBBX Family Genes in Various Organs and Their Possible Functions

**Figure 4:**
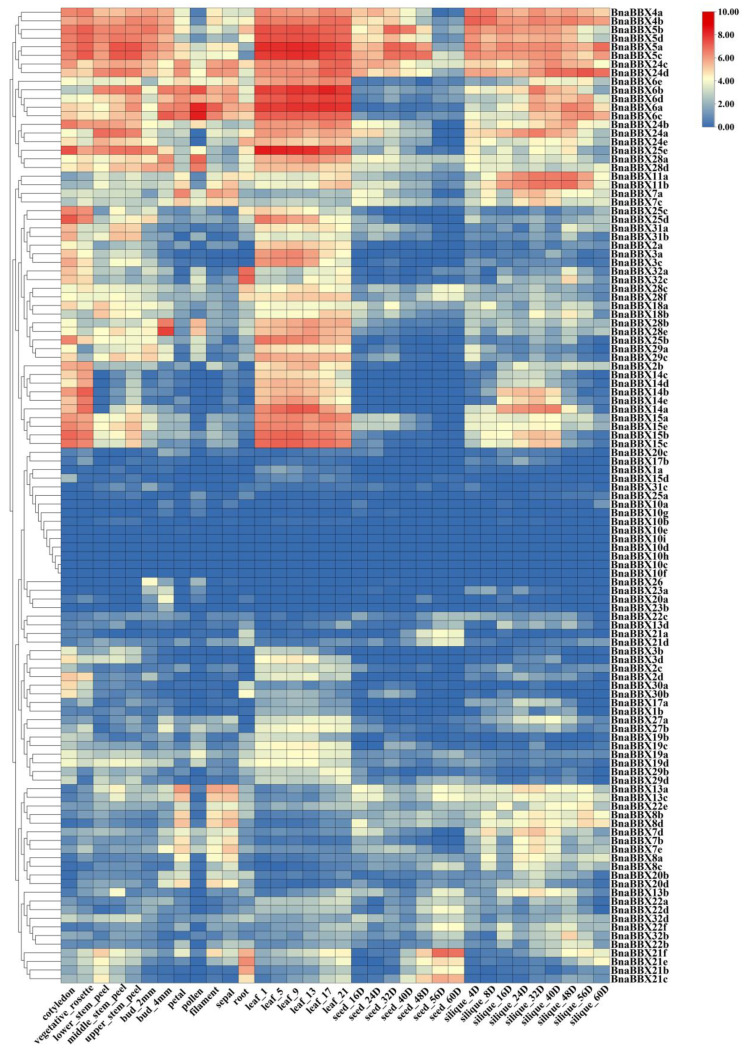
Heatmap of the expression patterns of *BnaBBXs* across different tissues and organs. The expression profiles of each *BnaBBX* gene are based on log2-transformed values (FPKM value + 1). FPKM, fragments per kilobase of exon model per million mapped fragments; DAF, days after flowering.

To investigate the potential functions of the BnaBBX family members, we examined their expression profiles. These genes are involved in all stages of development in *B. napus* and function in diverse organs, including bud, cotyledon, filament, leaf, stem peel, petal, pollen, root, seed, sepal, silique, and vegetative tissue (Figure 4 and Table S5). The tissue specificity index (TAU index) values of almost all BnaBBX genes were greater than 0.5, indicating that BnaBBX genes tend to be expressed in a specific manner rather than constitutively (Table S6). The expression profiles of these BBX family genes showed clear differences in expression among these tissues (with the exceptions of *BnaBBX10h* and *BnaBBX10i*, which were not expressed in any organ), suggesting that BnaBBX genes might play various roles in diverse organs and tissues. Furthermore, homologous BBX genes shared similar expression patterns. For example, *BnaBBX5a*, *BnaBBX5b*, *BnaBBX5c*, and *BnaBBX5d* were widely expressed in all tissues, especially in leaves and during the middle period of seed development. Both *BnaBBX14c* and *BnaBBX14d* were expressed at their highest levels in vegetative rosette tissue, while *BnaBBX22b* and *BnaBBX22e* were preferentially expressed during silique maturation. These results indicate that BBX family genes are widely involved in the processes of growth and development in *B. napus*.

### 2.6. BnaBBX Gene Promoters Contain Numerous Light-Responsive Cis-Elements

To further explore the functions of BnaBBX genes in plant responses to light, we analyzed the cis-regulatory elements within the 2000-bp regions upstream of the transcription start sites of these genes. We identified 29 cis-acting elements associated with light responses as well as circadian rhythms (Figure S12). The AE box, box 4, G box, GT1 motif, and TCT motif were widely present in the promoter regions of most BnaBBX genes. Moreover, BnaBBX family genes contained a minimum of 5 and a maximum of 26 light-responsive elements in their promoter regions, suggesting that BBX family members are closely associated with the light signal response in *B. napus*.

### 2.7. Expression Profiles of BnaBBX Genes during Seed Development under Shading Treatment

**Figure 5:**
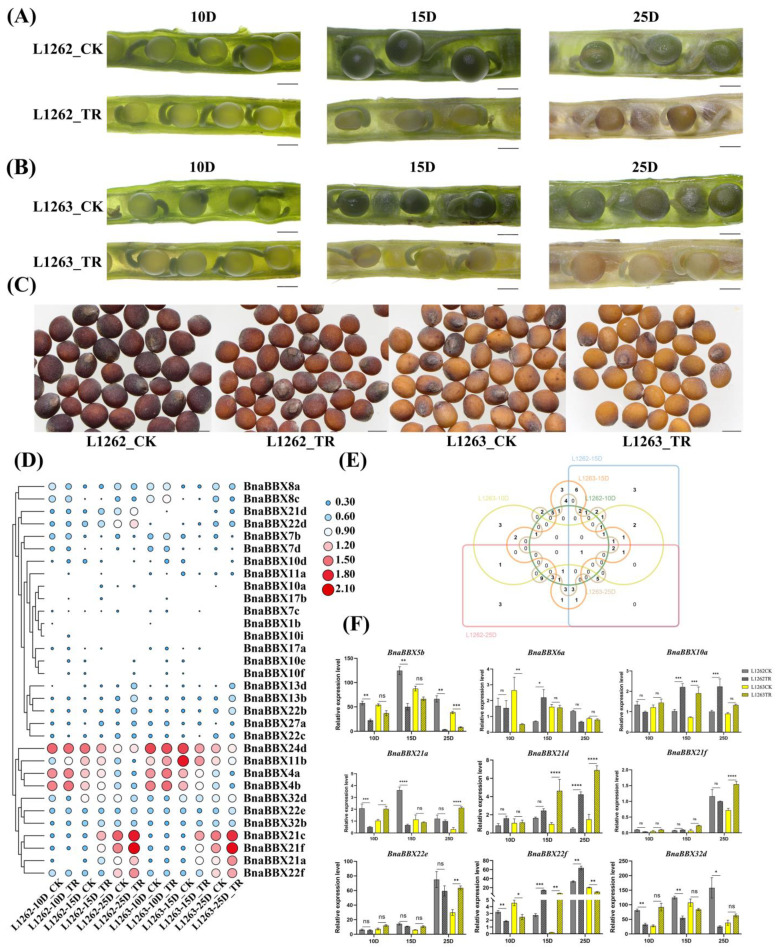
Expression patterns of BnaBBXs under shading conditions. (**A**,**B**) Phenotypes of L1262 (**A**) and L1263 (**B**) seeds under shading conditions (TR) and control conditions (CK, seeds under normal conditions) at different time points. D, days after shading. Scale bars, 2 mm. (**C**) Phenotypes of mature L1262 and L1263 seeds under shading conditions and the corresponding controls. Scale bars, 2 mm. (**D**) Heatmap of RNA-seq data of BnaBBX genes of seeds under shading. Three biological replicates for each type of sample were used. The transcriptome data of each *BnaBBX* gene are based on log_2_-transformed values (FPKM value + 1); (**D**) days after shading. (**E**) Venn diagram of genes with significant changes in expression (TR/CK, fold change ≥ 2 or fold change ≤ 0.5) before and after shading. (**F**) qRT-PCR analysis of BnaBBX family genes. The expression level of BnaActin7 was used to normalize the qRT-PCR data. *p*-values were calculated using multiple Student *t*-tests, comparing the levels in L1262CK (black-seeded material) and L1263CK (yellow-seeded material). * *p* < 0.05; ** *p* < 0.01; *** *p* < 0.001; ****p < 0.0001; ns, no difference. Error bars denote the standard deviation (SD) from three independent biological replicates. D, days after shading; TR, shading treatment.

To further elucidate the role of BnaBBXs in seed and silique development in response to light signals, we wrapped *B. napus* siliques with aluminum foil to shade them and observed the phenotypic changes in siliques exposed to varying durations of shading. Seeds grown under shaded conditions matured earlier and had a lighter pod color than the control. In addition, pigment accumulation decreased in mature seeds under shading treatment (Figure 5A–C). To investigate the roles of BnaBBXs in seed development, we used transcriptomic data to investigate the differences in their expression profiles under shaded conditions. 77 *BnaBBX* genes exhibited significant differences in expression levels under pre- vs. post-shading conditions among the 113 *BnaBBX* genes examined (Figure 5D,E and Figure S13). These changes were observed across various durations of shading treatment and in both yellow- and black-seeded materials, indicating that BnaBBX proteins play a crucial role in the light signaling pathway in *B. napus* seeds. However, the effects of shading treatment on *BnaBBXs* varied in yellow- vs. black-seeded materials (Figure 5E).

We measured the expression levels of eight randomly selected BBX genes that showed preferential expression in seeds or siliques, as well as one *BnaBBX5b* gene with high expression in seeds, using qRT-PCR to verify the reliability of the transcriptome data. *BnaBBX6a* was significantly upregulated in black-seeded materials after 25 days of shading, whereas no notable changes occurred in yellow-seeded materials before vs. after shading treatment (Figure 5D,F). Under shading treatment, the response patterns of *BnaBBX21d* in yellow- and black-seeded materials were markedly different. Moreover, the expression level of *BnaBBX22e* increased in the yellow-seeded line after 25 days of shading, whereas there was no significant change in expression level before vs. after shading treatment in the black-seeded line. The expression level of *BnaBBX32d* in the black-seeded but not the yellow-seeded line significantly increased after 15 or 25 days of shading treatment. These findings demonstrate that BnaBBX family genes serve as key regulators in seeds under shading conditions and that the biological functions of some BnaBBXs vary between yellow- and black-seeded materials.

### 2.8. Validation of Protein–Protein Interactions of BnaBBX22 and BnaA10.HY5

**Figure 6:**
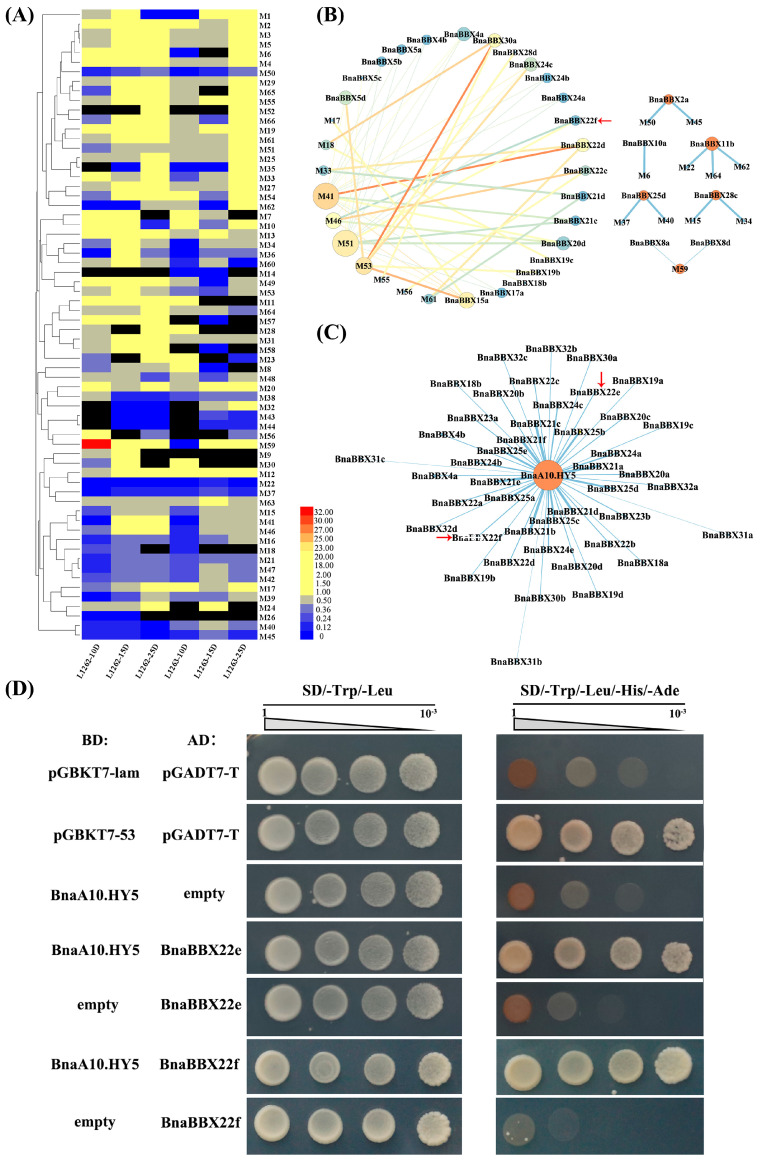
Validation of protein–protein interactions between BnaBBX22e and BnaA10.HY5. (**A**) Changes in metabolite contents before and after shading. Black blocks indicate that data were not available. The color scale represents the fold change in metabolite contents before and after shading, with red, blue, and yellow blocks indicating fold change ≥ 2, fold change ≤ 0.5, and fold change > 0.5 and <2, respectively. (**B**) The relationships among BnaBBX proteins and metabolites (|R value| ≥ 0.8, *p*-value ≤ 0.05). (**C**) Protein–protein interaction networks among BnaBBX proteins and BnaA10.HY5 proteins. The two red arrows point to BnaBBX22e and BnaBBX22f. (**D**) Yeast point-to-point validation between BnaBBX22e and BnaA10.HY5 as well as between BnaBBX22e and BnaA10.HY5. pGBKT7-53 and pGADT7-T, positive control; pGBKT7-lam and pGADT7-T, negative control; empty pGBKT7 and prey vector, testing for autoactivation and toxicity; bait vector and empty pGADT7, testing for autoactivation and toxicity.

Significant differences in pigment accumulation were visible in both yellow- and black-seeded materials before and after shading treatment (Figure 5C). We identified sixty flavonoids and six phenolic acids that might be associated with this treatment and analyzed the differential metabolites between control and shade-treated seeds. We observed a fold change ≥2 or ≤0.5 in the contents of forty-nine flavonoids and five phenolic acids pre- and post-shading. We also investigated the correlation between 77 differentially expressed BnaBBX genes and 54 differentially accumulated metabolites before and after shading, as shown in Figure 4 and Figure 6A. The expression pattern of *BnBBX22f* was closely related to the accumulation patterns of two flavonol derivatives, M46 and M51, before and after shading (Figure 6B, Tables S7 and S8). These results point to a strong link between the gene expression patterns and metabolic changes observed upon shading treatment.

Based on their preferential expression, distinct light responses between yellow- and black-seeded materials, and correlation between gene expression and metabolite profiles, we selected *BnaBBX22e* and *BnaBBX22f* for further investigation. We identified potential interactions between BnaBBX22e and BnaHY5, as well as between BnaBBX22f and BnaHY5, using the online tool STRING (Figure 6C). After analyzing the sequences and expression levels of five *BnaHY5* homologous genes (Figure S14), we constructed the *BnaA10.HY5* Bait vector to use in yeast two-hybrid screening. We also inserted the full-length coding sequences of *BnaBBX22e* and *BnaBBX22f* individually into the multiple cloning site (MCS) of the prey vector pGADT7. Co-transformation into yeast strain Y2HGold revealed protein–protein interactions of BnaBBX22e and BnaBB22f with BnaA10.HY5 (Figure 6D).

## 3. Discussion

In gene mining and mapping studies of genes associated with flowering time in rapeseed (*Brassica napus* L., AACC, 2n = 38), numerous BBX family genes have been identified, such as *BnaBBX1a* (a homolog of *AtBBX1*/*AtCO*) and *BnaBBX2b* (a homolog of *AtBBX2*/*AtCOL1*) [45,46,47]. However, little is known about BBX family genes in *Brassica* U-triangle crops, prompting us to perform a thorough investigation of the functions of BBX genes in these important crops.

In this study, we identified 482 BBX genes from the six *Brassica* U-triangle species. Despite variation in the number of BBX family members among *Brassica* species, the counts in these genes in *B. juncea* (AABB, 108), *B. napus* (AACC, 114), and *B. carinata* (BBCC, 96) are approximately equal to the combined total in their diploid progenitors: *B. rapa* (AA, 52) plus *B. nigra* (BB, 56), *B. rapa* (AA, 52) plus *B. oleracea* (CC, 56), and *B. nigra* (BB, 56) plus *B. oleracea* (CC, 56). The variation in the number of BBX family members among *Brassica* U-triangle species might be related to species-specific duplications or deletions that occurred during evolution. Moreover, the investigation of the Ka/Ks ratio indicated that purifying selection has been present in most BBXs over evolutionary time, maintaining high collinearity among the corresponding chromosomes following their diverging from a common ancestor (Tables S2–S5).

We also investigated the phylogenetic relationship and conserved domains of the entire BBX gene family in *Brassica* U-triangle species. Based on their conserved domains, our analysis divided the BBX proteins in *Brassica* into five groups, aligning generally with previous categorizations of BBX proteins in *Arabidopsis*, rice, tomato (*Solanum lycopersicum*), and 12 other plant species [48,49,50]. The conserved number of groups in different species suggests that BBXs might function in essential physiological processes involved in plant growth and development. *Brassica* BBX proteins in Groups I, II, and IV possess the B-box1 and B-box2 domains, whose amino acid sequences share high similarities among BBX proteins encoded in the A, B, and C subgenomes in the six *Brassica* species. These findings indicate that BBX genes from six *Brassica* species might have high functional similarities during the evolution. Moreover, the majority of BBX proteins within these groups that lack structural domains were identified in allotetraploid plants (*B. juncea*, *B. napus*, and *B. carinata*), with a smaller proportion identified in diploid plants (*B. rapa*, *B. nigra*, and *B. oleracea*). These observations indicate that duplication events of BBX genes have occurred, during which some genes have undergone segmental deletions.

BBX proteins play critical roles in various biological processes, including the shade-avoidance response and circadian rhythms [1,51]. In this study, we revealed that various cis-acting regulatory elements involved in light response are widely distributed in the promoter regions of BnaBBX genes, including the G box, box 4, and box I. These findings indicate that BBX family genes in *B. napus* are widely involved in light signaling pathways. *BnaBBX3d*, *BnaBBX6a*, and *BnaBBX6b* are located in a candidate genomic region thought to be closely related to flowering time based on differences in sequence annotations, resequencing data, and genome-wide association study (GWAS) [52,53]. Transcriptome data indicate that both *BnaBBX6a* and *BnaBBX6b* are highly expressed in buds, sepals, filaments, and petals, which is highly consistent with the above findings (Figure 4). In addition, BBX7 and BBX8 play pivotal roles in regulating flowering in Arabidopsis and *chrysanthemum* [54,55]. Homologs of *AtBBX7* and *AtBBX8* in B. napus were preferentially expressed in floral organs, suggesting that they might regulate flower development. These findings suggest that BBX genes in rapeseed share functional similarities with their homologs in other species, implying they play conserved roles in the light signaling pathway across species.

BBX family genes are involved in the light-induced biosynthesis of the flavonoids among various plant species [36,56,57]. Flavonoid biosynthesis in *B. napus* is closely related to the formation of seed coat color [58,59]. Our study uncovered significant differences in the shade-responsive expression patterns of BnaBBX genes (including *BnaBBX6a*, *BnaBBX22e*, and *BnaBBX32d*) between yellow-seeded and black-seeded materials, concomitant with changes in the accumulation patterns of various flavonoid compounds under shading conditions. Transcriptomic-metabolomic correlation analysis under shading conditions revealed a significant association between the expression dynamics of the BnaBBX gene family and fluctuations in metabolite levels, particularly in the case of *BnaBBX22f* and two flavonoid metabolites, M46 and M51 (Figure 6B). M46 and M51 are intermediate compounds in the flavonoid pathway, potentially serving as color-developing compound precursors. Collectively, these findings suggest that changes in gene expression might be responsible for the observed differences in metabolic profiles under different light conditions. Finally, BBX22 interacts with HY5, potentially upregulating the transcription of genes related to flavonoid biosynthesis regulated by HY5 [35,60]. BnaBBX22e and BnaBBX22f undergo protein–protein interactions with BnaA10.HY5 (Figure 6D). Together, these findings suggest that BnaBBXs function in flavonoid accumulation in seeds under shading conditions, thereby affecting seed coat color.

## 4. Materials and Methods

### 4.1. Plant Materials and Sample Collection

The rapeseed (*Brassica napus*) varieties L1262 (black-seeded rapeseed) and L1263 (yellow-seeded rapeseed) were planted as previously described [61]. The siliques were fully covered with aluminum foil 15 days after flowering (15 DAF); uncovered siliques on the opposite side of the branch were used as controls. After 10 days, 15 days, and 25 days of shading treatment, fresh seeds were collected from ten plants of the same cultivar that had undergone the same duration of treatment, immediately put into liquid nitrogen, and preserved at −80 °C for RNA extraction.

### 4.2. Identification and Characterization of BBX Family Proteins

The protein sequences of AtBBXs were retrieved from The Arabidopsis Information Resource (TAIR10) database (https://www.Arabidopsis.org/ (accessed on 14 February 2024) and served as queries for a BLASTp search using data of *Brassica* U-triangle species (*B. rapa*, *B. nigra*, *B. oleracea*, *B. juncea*, *B. napus*, and *B. carinata*) obtained from the *Brassica* Database (BRAD, http://Brassicadb.cn) and the *Brassica napus* multi-omics information resource database (BnIR, https://yanglab.hzau.edu.cn/BnIR (accessed on 14 February 2024))) [62,63,64]. Target genes identified by TBtools-BLAST were confirmed using the National Center for Biotechnology Information (NCBI) Batch CD-SearchTool (https://www.ncbi.nlm.nih.gov/Structure/bwrpsb/bwrpsb.cgi (accessed on 20 February 2024)), BRAD-Syntenic Gene @ subgenomes (http://Brassicadb.cn/#/syntenic-gene/ (accessed on 20 February 2024)), and the Quick Find Best Homology module in TBtools [65,66]. The online tool Cell-PLoc 2.0 (http://www.csbio.sjtu.edu.cn/bioinf/plant-multi/) was used to predict the subcellular localizations of the proteins [67]. The molecular weight (MW), isoelectric point (pI), and grand average of hydropathy (GRAVY) value of each BBX protein were also investigated utilizing the TBtools module Protein Parameter Calc [66].

### 4.3. Multiple Sequence Alignment and Phylogenetic Analysis

To determine the evolutionary relationships of the BBXs, the predicted amino acid sequences of the BBX proteins from six different *Brassica* species were analyzed using multiple sequence alignment and phylogenetic analysis. Multiple sequence alignment was conducted using MEGA 11 under the default mode [68]. All phylogenetic trees presented in this article were constructed using the maximum-likelihood (ML) method with IQ-TREE software (version 2.1.4-beta COVID-edition for Linux 64-bit, built on Jun 24 2021) [69] and analyzed by bootstrap analysis using 1000 replicates and the Le and Gascuel (LG) matrix as the best-fit model. All trees were further mapped using the website Interactive Tree of Life (version 6.9) (iTOL, https://itol.embl.de/ (accessed on 29 February 2024)) [70]. The BnaBBXs were named based on their phylogenetic relationships with AtBBXs, following a consistent nomenclature.

### 4.4. Identification of Conserved Motifs, Conserved Domain Searches, and Gene Structure Analysis

MEME online (http://meme-suite.org/tools/meme (accessed on 2 March 2024)) was used for identifying the conserved motifs of BBX proteins with default parameters, except for setting the maximum number of predicted motifs to 10 [71]. Conserved domains were identified using the online programs NCBI (https://www.ncbi.nlm.nih.gov/Structure/bwrpsb/bwrpsb.cgi (accessed on 5 March 2024)), InterPro (http://www.ebi.ac.uk/interpro/search/sequence/ (accessed on 7 March 2024)), and HMMER (https://www.ebi.ac.uk/Tools/hmmer/ (accessed on 9 March 2024)) [65,72,73]. Exon–intron structures for Brassica BBXs were extracted from the *Brassica* U-triangle species gene information GFF3 file (obtained from BRAD, http://Brassicadb.cn (accessed on 14 February 2024)) and then mapped using TBtools software (v 2.070) (https://github.com/CJ-Chen/TBtools (accessed on 10 March 2024)) [64,66].

### 4.5. Collinearity Analysis of Brassica BBX Genes

The analysis of Gene synteny was performed using TBtools-One Step MCScanX (Guangzhou, GD, China) with default parameters. The synteny maps of the orthologous BBX family genes from *B. napus* and other selected species were constructed with the Advanced Circos module in TBtools [74]. Additionally, both the nonsynonymous (Ka) and synonymous (Ks) substitutions for each BBX pair and the genome-wide gene density distributions in the genomes of all six Brassica species were calculated using TBtools [66].

### 4.6. Expression Profiling of BnaBBX Family Genes

RNA-seq data were sourced from BnIR (https://yanglab.hzau.edu.cn/BnIR (accessed on 14 March 2024)) to explore the expression specificity of *Brassica* BBX genes across different tissues [63]. The data were converted into log_2_ values (FPKM value + 1) to better visualize and compare expression profiles. Furthermore, tissue specificity index (TAU index) calculation in TBtools was used to determine the preferential expression pattern of each gene [63,66].

Total RNA was isolated from the samples with a DNAaway RNA Mini-prep Kit (Sangon Biotech, Shanghai, China). After examining the quantity and quality of the purified RNA, a library was constructed using standard protocols and sequenced on the Illumina HiSeq2000 sequencing platform (Tianjin Novogene Bioinformatic Technology Corporation, Ltd., Tianjin, China). Transcriptome analysis was then performed as described previously [61]. To investigate gene expression patterns in siliques in response to shading, the BnaBBX gene expression profiles were further clarified using the RNA-seq dataset under shading conditions. Heatmaps were generated using TBtools to display *Brassica* BBX gene expression, and the data were converted into log_2_ values (FPKM value + 1) to better present the expression profiles [66].

Total RNA obtained before was used for qRT-PCR. The synthesis of first-strand complementary and qRT-PCR analysis was conducted as previously described, and we normalized the results to the reference gene *BnaActin7* (EV116054) using the 2^−∆∆Ct^ method [39,61]. Each sample in this study had three biological replicates. The primers are displayed in Table S8. Statistical significance was determined via Student *t* -tests; *p*-values of ≤0.05 and ≤0.01 were deemed to be significant and highly significant, respectively.

### 4.7. Analysis of Light-Responsive Elements in the BnaBBX Gene Promoters

The 2000-bp region upstream from the start codon of each BnaBBX gene was extracted from the ZS11.V0 genome (obtained from BnIR, https://yanglab.hzau.edu.cn/BnIR (accessed on 14 February 2024)) using TBtools-Gtf/Gff3 Sequences Extract [63,66]. Subsequently, the promoter sequence was uploaded to the PlantCARE website (http://bioinformatics.psb.ugent.be/webtools/plantcare/html/ (accessed on 16 March 2024)) to predict putative cis-acting elements [75]. Functional annotation of light-responsive elements was conducted through statistical analysis of their types and quantities.

### 4.8. Flavonoid Extraction and UPLC-HESI-MS/MS Analysis

Flavonoids were extracted from seed coats (100 mg fresh weight) stored at −80 °C and subjected to UPLC-HESI-MS/MS analysis. Retention times, relative molecular masses, mass spectra, and information from public databases were used to identify flavonoid metabolites [39,76]. The contents of flavonoid metabolites were determined using standard curves obtained from epicatechin, quercetin, kaempferol, and isorhamnetin (Sigma Aldrich, Shanghai, China). All analyses were conducted in triplicate.

### 4.9. Yeast Two-Hybrid Point-to-Point Validation of BnaBBX22e and BnaA10.HY5

Primers were designed based on the genome data for ZS11.V0 (BnIR, https://yanglab.hzau.edu.cn/BnIR (accessed on 14 February 2024)) using SnapGene 4.1.9 (https://www.snapgene.com/ (accessed on 24 March 2024)) [63]. PCR was performed in a 50 µL reaction volume using cDNA from mixed ZS11 seeds as the template based on the manual for the 2× TransStart^®^ FastPfu PCR SuperMix (+dye) (TransGen Biotech, Beijing, China). Following the manufacturer’s instructions, PCR was performed at 95 °C for 2 min, 95 °C for 20 s, 56 °C for 20 s, 72 °C for 40 s (35 cycles), and 72 °C for 5 min. The PCR product was inserted into the pEASY^®^-Blunt Simple Cloning Vector (provided by TransGen Biotech, Beijing, China) for sequencing. The primers used are listed in Table S6.

Following the Matchmaker GAL4-based two-hybrid assay, the full-length coding sequences (CDS) of *BnaBBX22e* and *BnaBBX22f* were separately inserted into the NC Frame of prey vector pNC-GADT7, while *BnaA10. HY5* protein is expressed as a fusion with the Gal4 DNA-binding domain (GAL4-BD, pNC-GBKT7) [77]. The PEG/LiAc-based method was utilized to co-transform yeast strain Y2HGold with the bait and prey constructs. Following transformation, the yeast cells were cultured on double dropout (DDO, SD/–Trp/–Leu) medium and cultured at 30 °C for a period of 3 to 5 days. A single plaque was resuspended in 100 µL of sterilized 0.9% NaCl, and 10 µL samples of a 1/10 dilution, a 1/100 dilution, and a 1/1000 of the transformation mixture were spotted onto quadruple dropout (QDO, SD/–Trp/–Leu/–His/–Ade) plates. Yeast growth was observed following incubation at 30 °C for 3–5 days.

## 5. Conclusions

In our study, 482 BBX family genes were identified from *Brassica* U-triangle species and classified into five groups based on their domain composition. Among all six *Brassica* species, the proteins in the same branches of the polygenetic trees shared similar functional structures and domains. The expression profiles in siliques under shading treatment revealed that the BnaBBXs are strongly associated with light responses. Moreover, numerous light-responsive cis-elements were identified in the promoter regions of the BnaBBX genes, supporting this conclusion. Metabolome analysis showed that the flavonoid metabolite contents strongly differed before and after shading and that these differences were correlated with the variation in BnaBBX gene expression. Yeast two-hybrid point-to-point validation between BnaBBX22e and BnaA10.HY5 as well as between BnaBBX22f and BnaA10.HY5 supported the possibility that BnaBBX22e and BnaBBX22f participate in light-induced anthocyanin accumulation in seeds. Overall, the findings obtained from this study establish the foundation for future functional analysis of *Brassica* BBX family members, especially elucidating their involvement in light-induced anthocyanin accumulation.

## Data Availability

All additional datasets supporting the findings of this study are included within the article and Supplementary Materials.

## Supplementary Materials

The following supporting information can be downloaded at https://www.mdpi.com/article/10.3390/plants13162226/s1, Figure S1: Phylogenetic tree of B-box genes from Arabidopsis and *Brassica* U-triangle species in A genome; Figure S2: Phylogenetic tree of B-box genes from *Arabidopsis* and *Brassica* U-triangle species in B genome; Figure S3: Phylogenetic tree of B-box genes from *Arabidopsis* and *Brassica* U-triangle species in C genome; Figure S4: The number of different groups of BBX proteins; Figure S5: The sequences of the 10 Motifs for Figure S6B, Figure S7B and Figure S8B; Figure S6: Conserved motifs and exon–intron structure of BBXs based on phylogenetic tree between Arabidopsis and U-triangle species in A subgenome; Figure S7: Conserved motifs and exon–intron structure of BBXs based on phylogenetic tree between *Arabidopsis* and U-triangle species in B subgenome; Figure S8: Conserved motifs and exon–intron structure of BBXs based on phylogenetic tree between *Arabidopsis* and U-triangle species in C subgenome; Figure S9: Syntenic relationships of BBX family genes among *B. rapa*, *B. junce* and *B. napus*; Figure S10: Syntenic relationships of BBX family genes among *B. nigra*, *B. juncea* and B. *carinata*; Figure S11: Syntenic relationships of BBX family genes among *B. oleracea*, *B. napus*, and *B. carinata*; Figure S12: Light responsive cis- elements analysis of BnaBBX genes promoters; Figure S13: Heatmap of the expression profiles of *BnaBBXs* for seed under shading condition; Figure S14: The expression profiles and sequences alignment analysis of BnaHY5 genes; Table S1: The physicochemical properties and the prediction of subcellular localization for BBX proteins; Table S2: The Ka/Ks ratio of BBX genes from A subgenome; Table S3: The Ka/Ks ratio of BBX genes from B subgenome; Table S4: The Ka/Ks ratio of BBX genes from C subgenome; Table S5: The transcriptome data of BnaBBX family genes in various organs; Table S6: The Tissue Specificity Index (TAU index) of BnaBBX family genes; Table S7: The detailed information and contents of the metabolites; Table S8: Correlation analysis of BnaBBX genes expression and metabolite profiles; Table S9: The Primer used in this paper.
