# Supplementary material for: Genome-Wide Identification of B-Box Family Genes and Their Potential Roles in Seed Development under Shading Conditions in Rapeseed
Source: Plants (Basel). 2024 Aug 11;13(16):2226. doi: 10.3390/plants13162226 (PMC11359083; doi:10.3390/plants13162226)
Supplement: Supplementary file 1 [file plants-13-02226-s001.zip › Supplementary Figure.pdf]

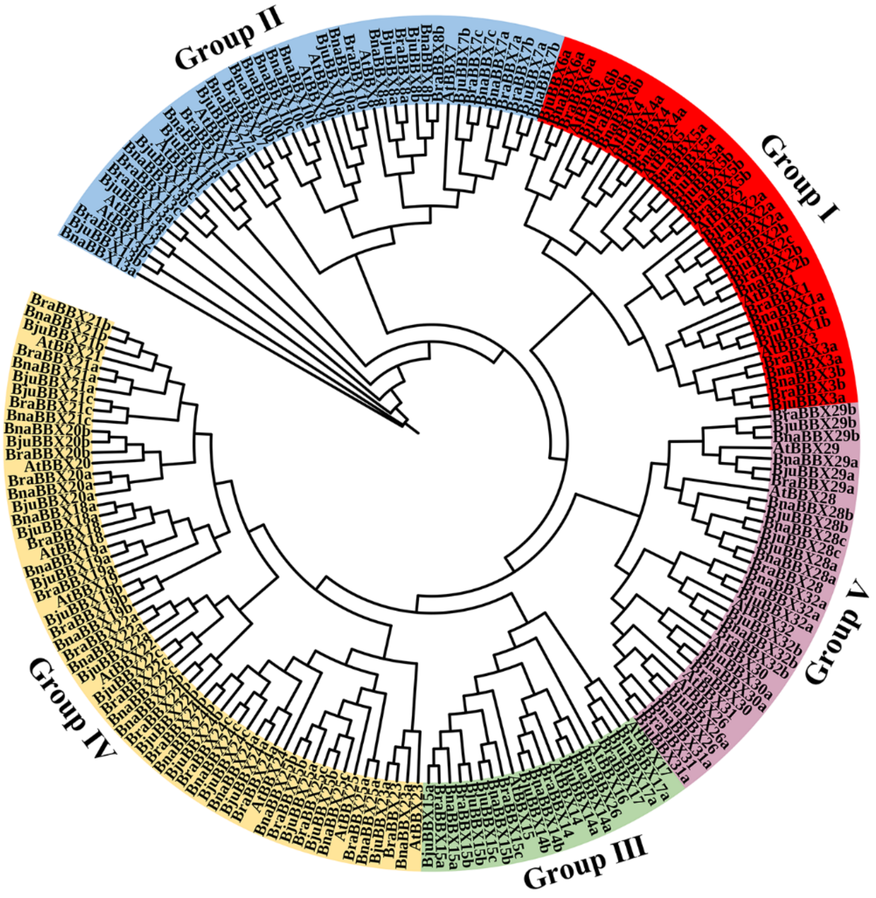

Figure S1. Phylogenetic tree of B-box genes from *Arabidopsis* and *Brassica* U-triangle species in A genome. The phylogenetic tree, which is based on the protein matrix using iq-tree, is divided into five clades (I-V) labeled with different colors. The phylogenetic tree was constructed using Arabidopsis, *Brassica rapa* (Bra, AA), *Brassica juncea* (Bju, AABB) and *Brassica napus* (Bna, AACC)

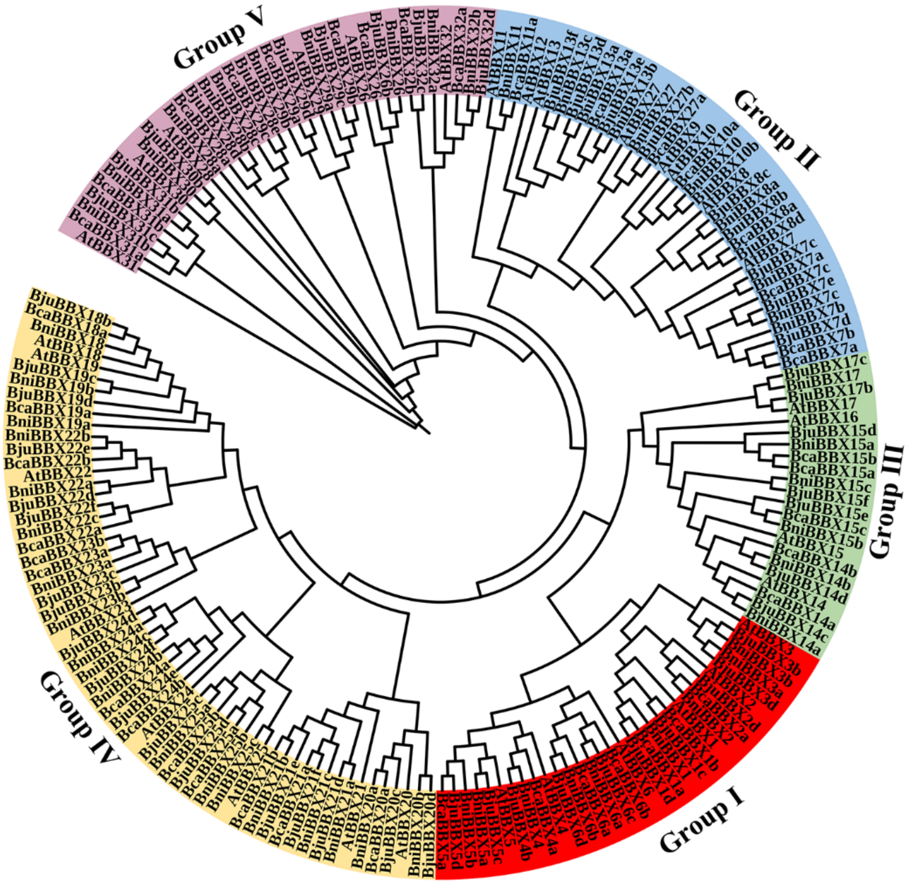

Figure S2. Phylogenetic tree of B-box genes from *Arabidopsis* and *Brassica* U-triangle species in B genome. The phylogenetic tree, which is based on the protein matrix using iq-tree, is divided into five clades (I-V) labeled with different colors. The phylogenetic tree was constructed using Arabidopsis, *Brassica nigra* (Bni, BB), *B. juncea* (Bju, AABB), *Brassica carinata* (Bca, BBCC).

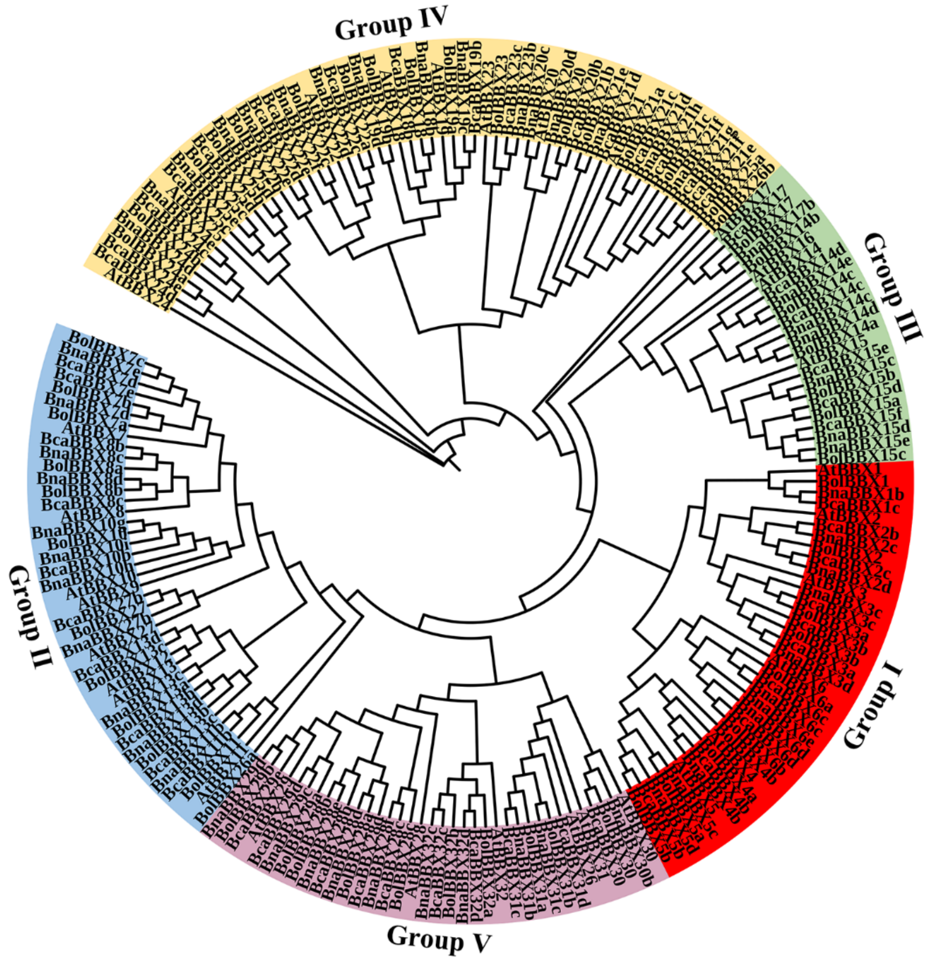

Figure S3. Phylogenetic tree of B-box genes from *Arabidopsis* and *Brassica* U-triangle species in C genome. The phylogenetic tree, which is based on the protein matrix using iq-tree, is divided into five clades (I-V) labeled with different colors. The phylogenetic tree was constructed using Arabidopsis, *Brassica oleracea* (Bol, CC), *B. napus* (Bna, AACC) and *B. carinata* (Bca, BBCC).

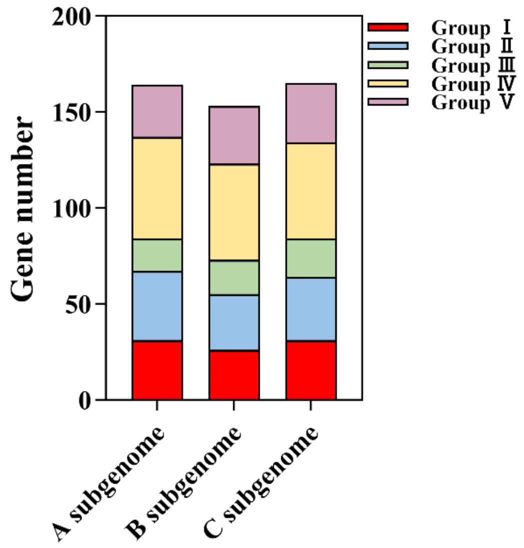

Figure S4. The number of different groups of BBX proteins.

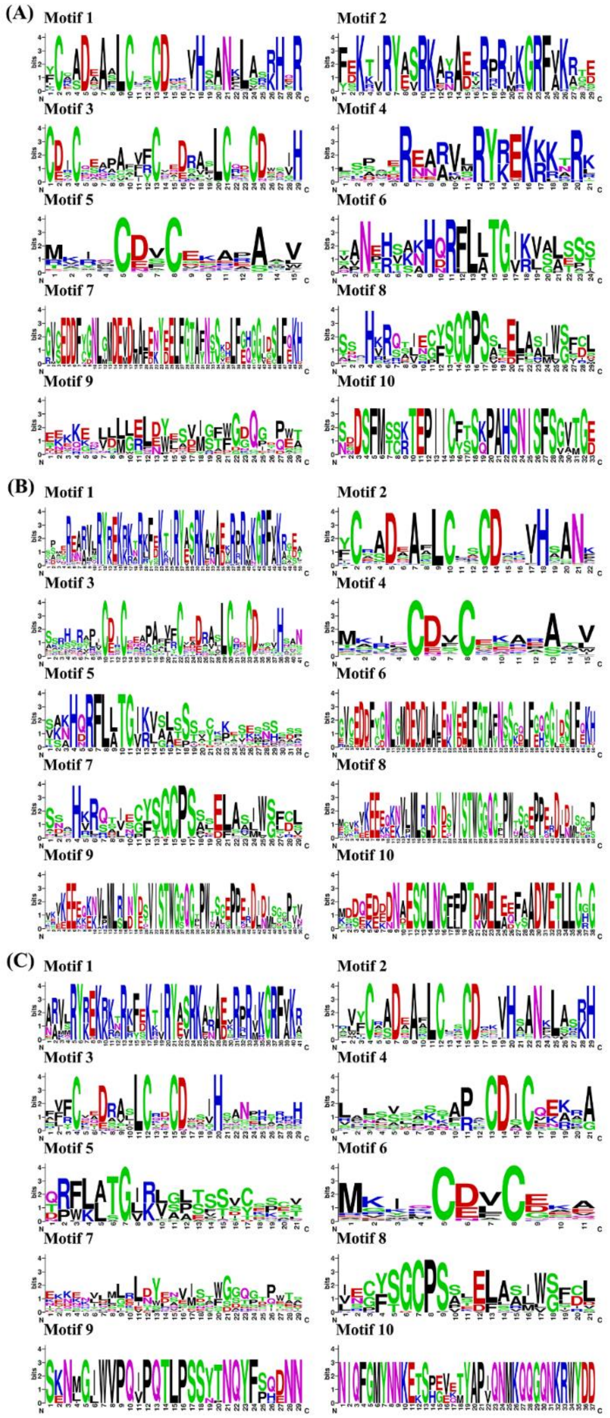

Figure S5. The sequences of the 10 Motifs for Figure S6B，Figure S7B and Figure S8B. (A, B, C) The sequences of conserved motif for BBX protein in A, B and C genome. The *x*-axis shows the conserved sequences of the structural domains, with the height of each letter indicating the level of conservation of each residue across all proteins. The *y*-axis represents the relative entropy scale, which reflects the conservation rate of each amino acid.

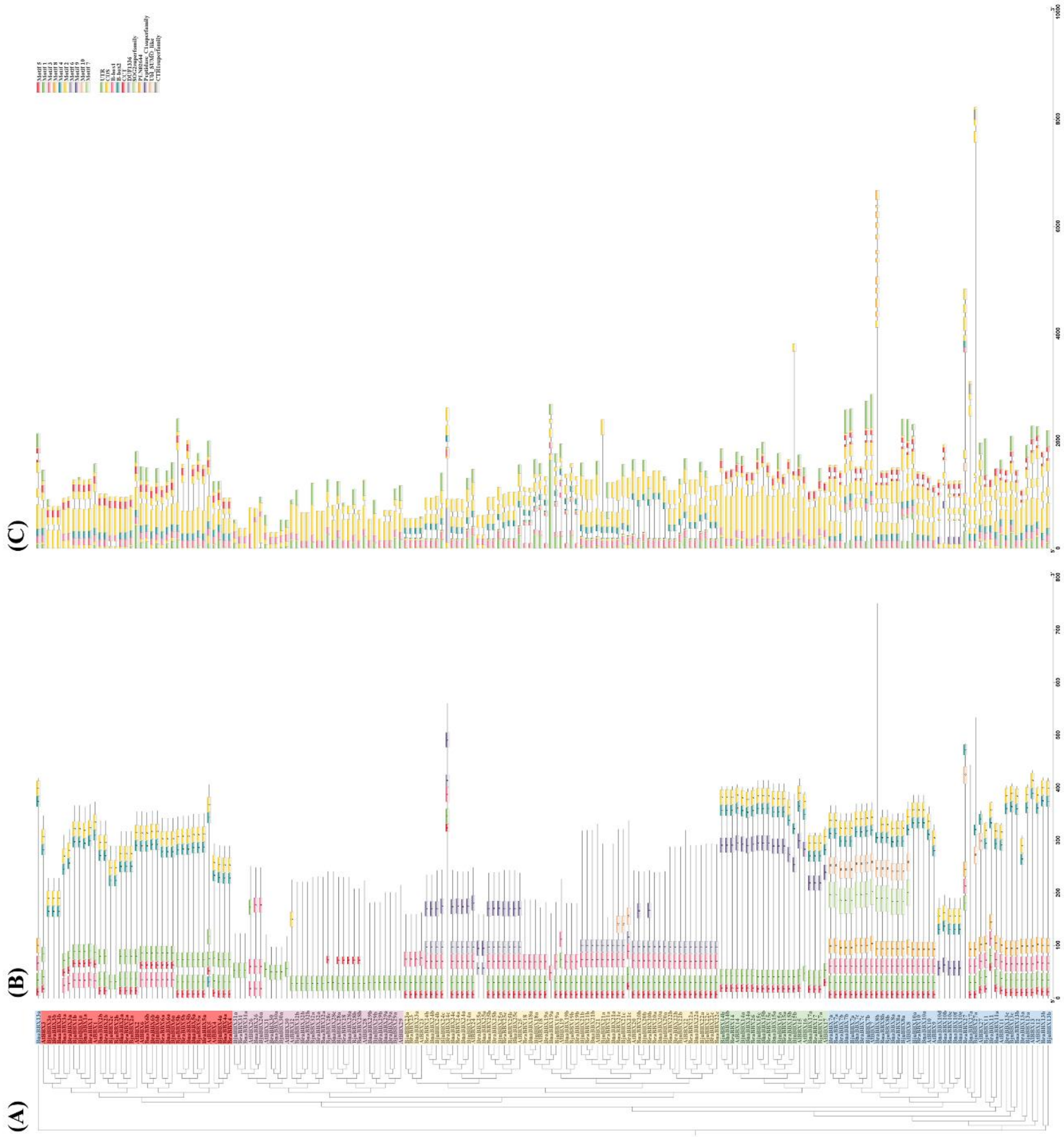

Figure S6. Conserved motifs and exon-intron structure of BBXs based on phylogenetic tree between *Arabidopsis* and U-triangle species in A subgenome. (A) Phylogenetic tree of the BBX family proteins from *Arabidopsis* and the *Brassica* U-triangle species (same with Figure S1). (B) The conserved motifs of the BBX proteins in A subgenome. The motifs, numbered 1-10, are displayed by rectangular boxes with different colors. (C) Conserved domains and exon-intron structure of BBX gene family in A genome. The green boxes indicate UTR, the yellow boxes indicate exon and the grey lines represent intron.

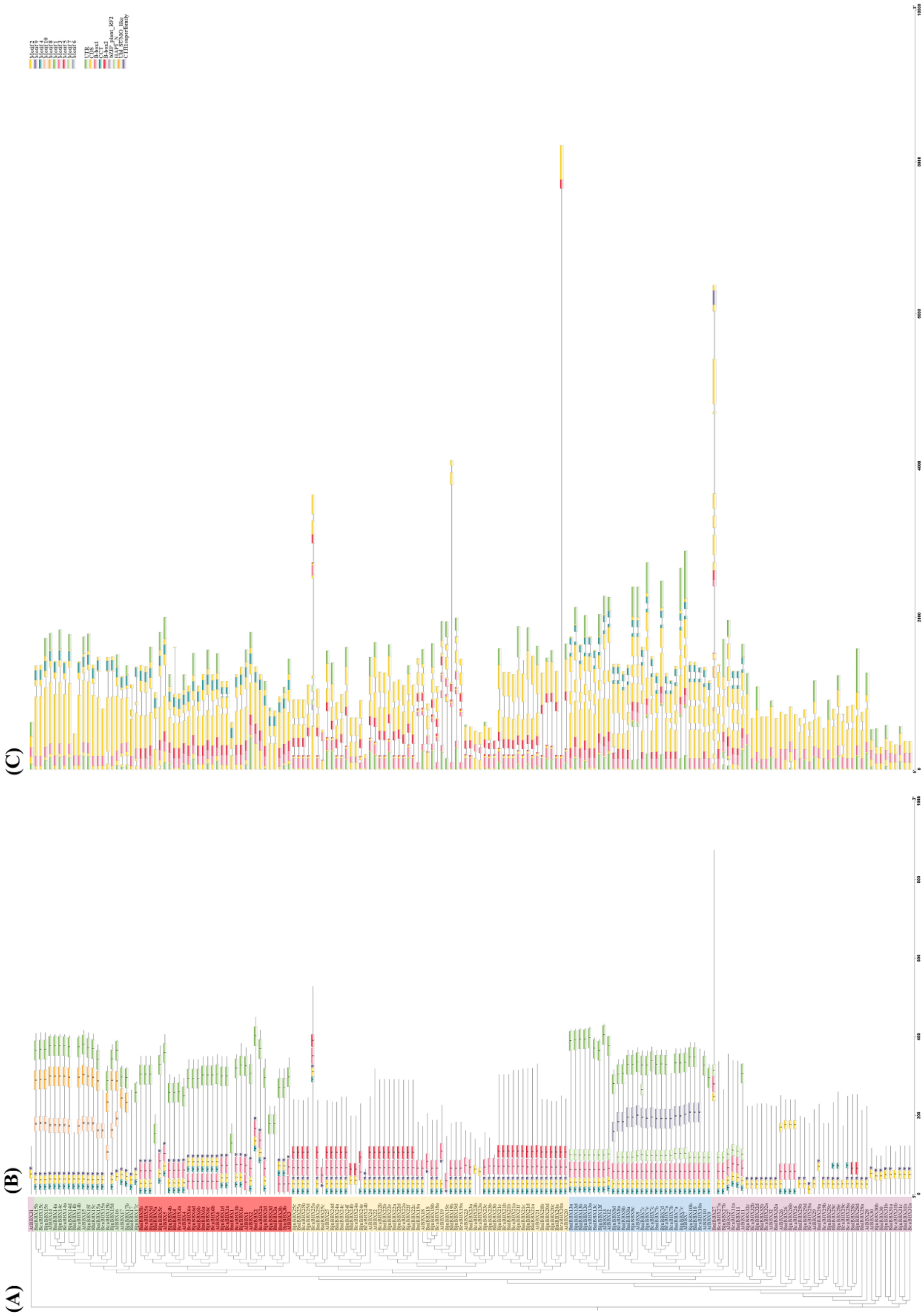

Figure S7. Conserved motifs and exon-intron structure of BBXs based on phylogenetic tree between *Arabidopsis* and U-triangle species in B subgenome. (A) Phylogenetic tree of the BBX family proteins from *Arabidopsis* and the *Brassica* U-triangle species (same with Figure S2). (B) The conserved motifs of the BBX proteins in B subgenome. The motifs, numbered 1-10, are displayed by rectangular boxes with different colors. (C) Conserved domains and exon-intron structure of BBX gene family in B genome. The green boxes indicate UTR, the yellow boxes indicate exon and the grey lines represent intron.

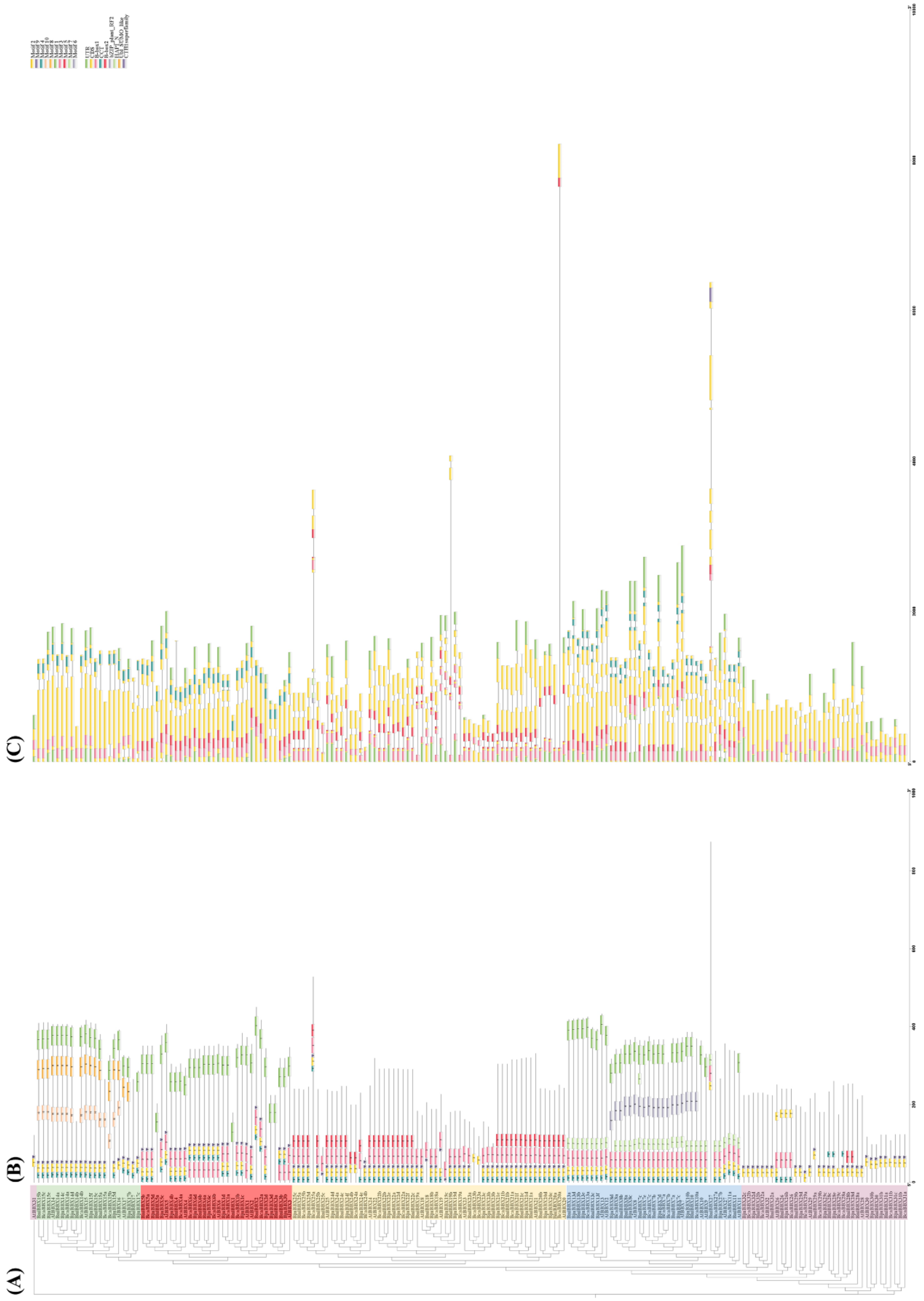

Figure S8. Conserved motifs and exon-intron structure of BBXs based on phylogenetic tree between *Arabidopsis* and U-triangle species in C subgenome. (A) Phylogenetic tree of the BBX family proteins from *Arabidopsis* and the *Brassica* U-triangle species (same with Figure S3). (B) The conserved motifs of the BBX proteins in B subgenome. The motifs, numbered 1-10, are displayed by rectangular boxes with different colors. (C) Conserved domains and exon-intron structure of BBX gene family in C genome. The green boxes indicate UTR, the yellow boxes indicate exon and the grey lines represent intron.

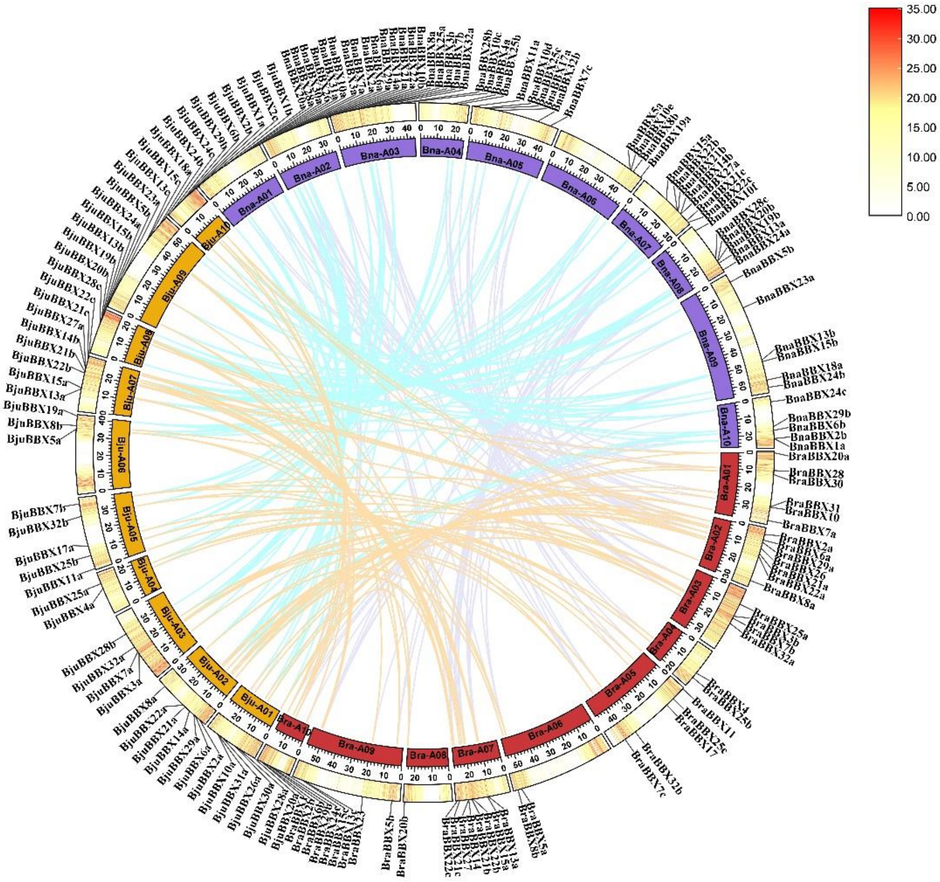

Figure S9. Syntenic relationships of BBX family genes among *B. rapa*, *B. junce* and *B. napus*. The chromosomes are shown in different colors from the *Brassica* U-triangle species. The syntenic regions are represented by different colored lines. The scales represent the length of the chromosomes.

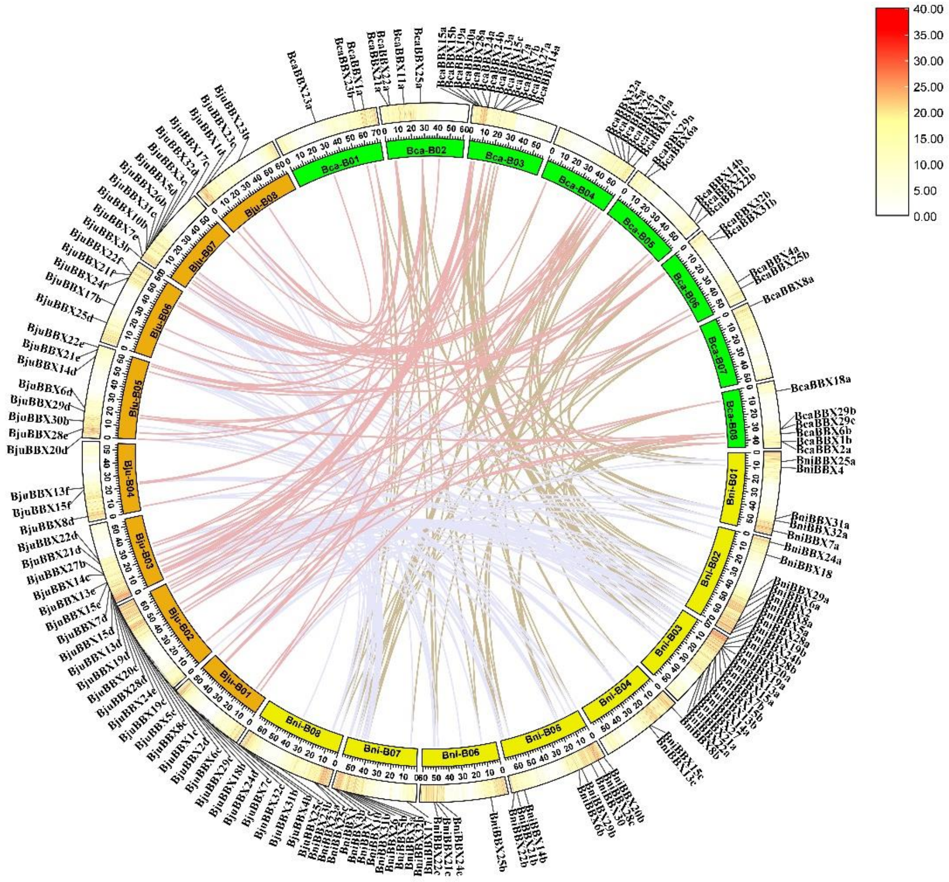

Figure S10. Syntenic relationships of BBX family genes among *B. nigra*, *B. juncea* and *B. carinata*. The chromosomes are shown in different colors from the *Brassica* U-triangle species. The syntenic regions are represented by different colored lines. The scales represent the length of the chromosomes.

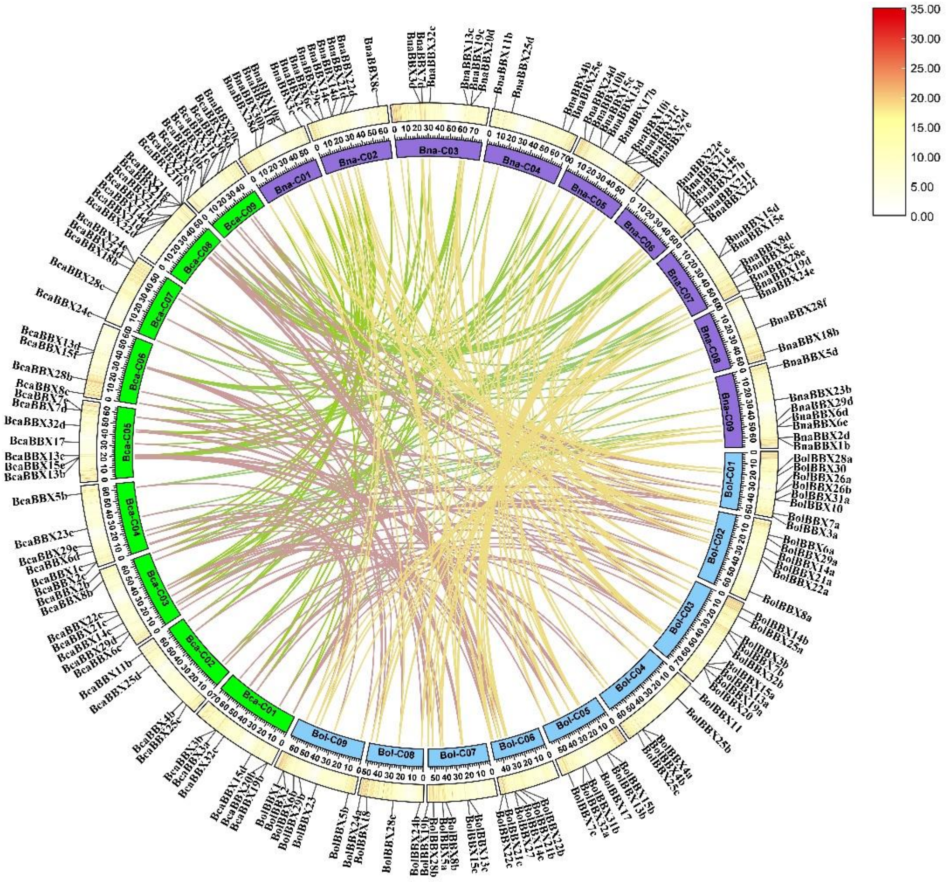

Figure S11. Syntenic relationships of BBX family genes among *B. oleracea*, *B. napus*, and *B. carinata*. The chromosomes are shown in different colors from the *Brassica* U-triangle species. The syntenic regions are represented by different colored lines. The scales represent the length of the chromosomes.

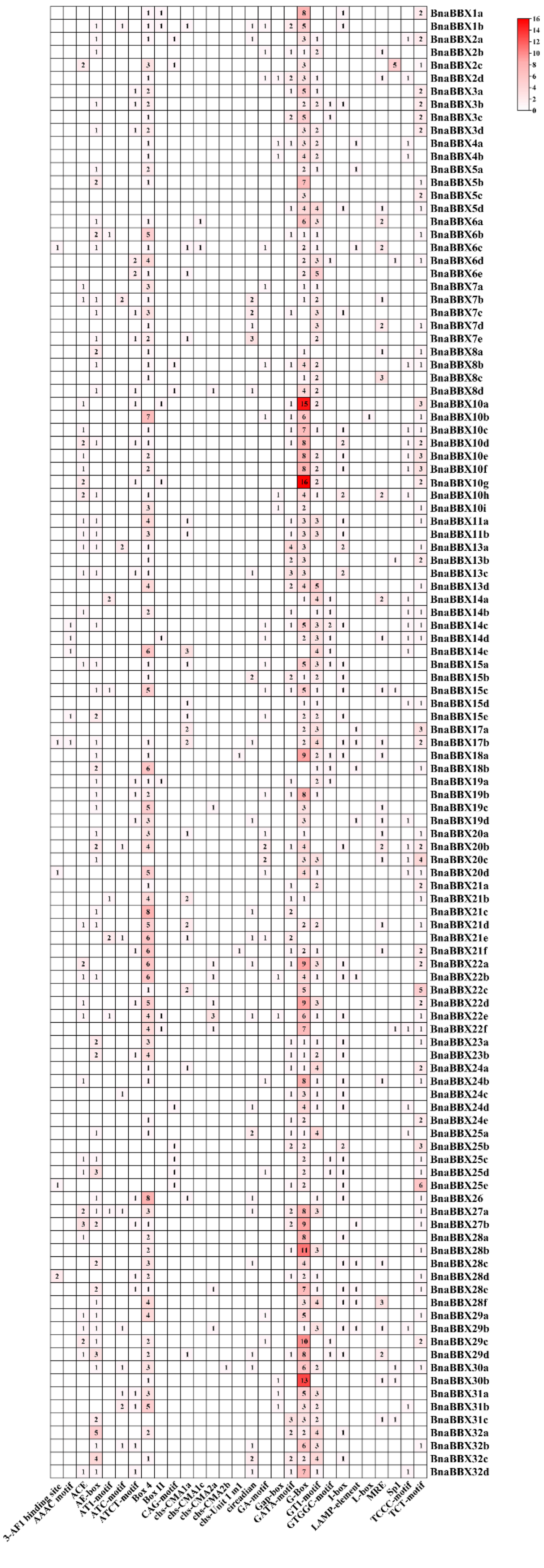

Figure S12. Light responsive cis- elements analysis of BnaBBX genes promoters. Counts were the number of cis-elements.

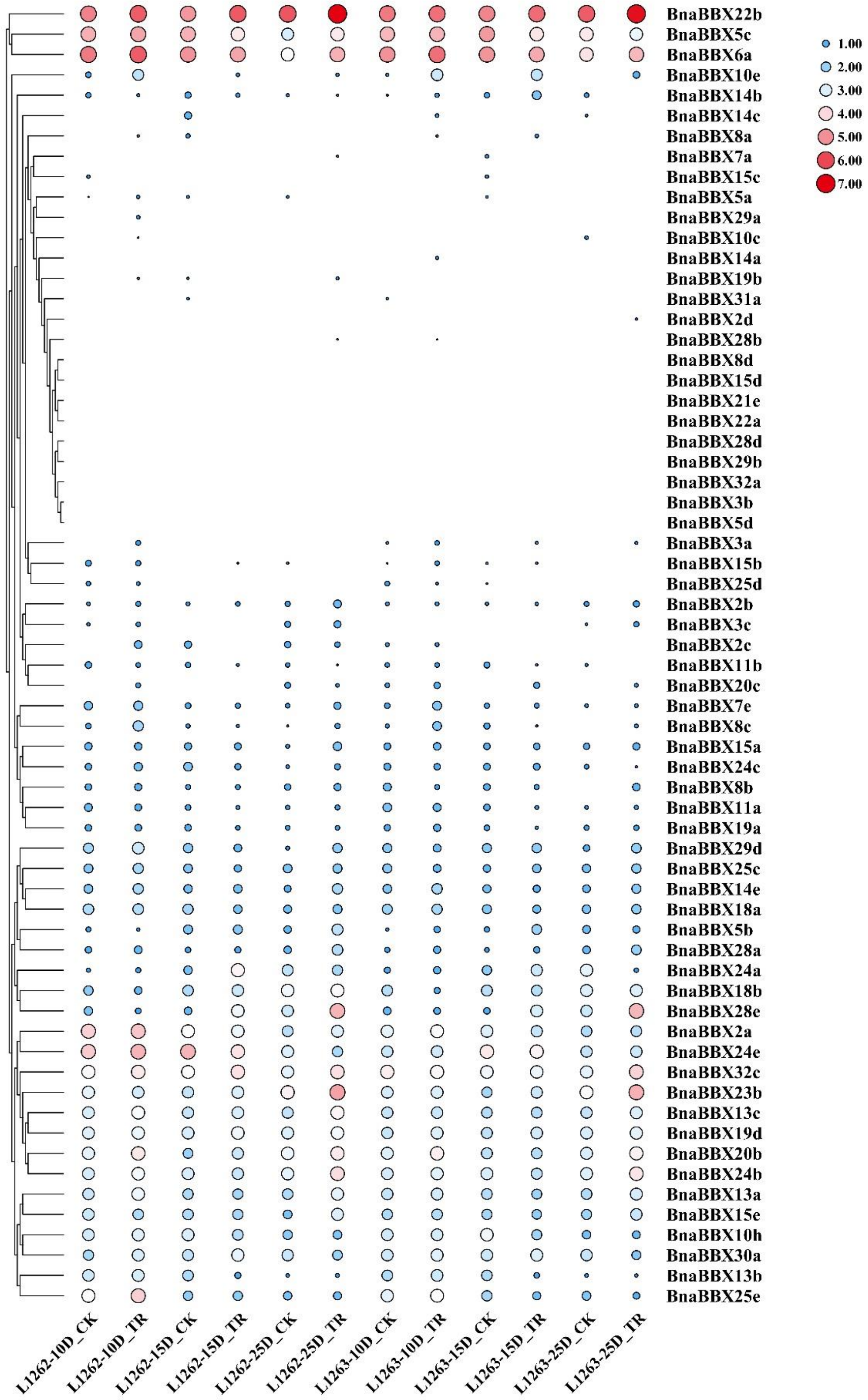

Figure S13. Heatmap of the expression profiles of *BnaBBXs* for seed under shading condition. The expression profiles of each BnaBBX genes are based on $\log_2$-transformed values (FPKM value + 1). FPKM, fragments per kilobase of exon model per million mapped fragments; D, days after shading.

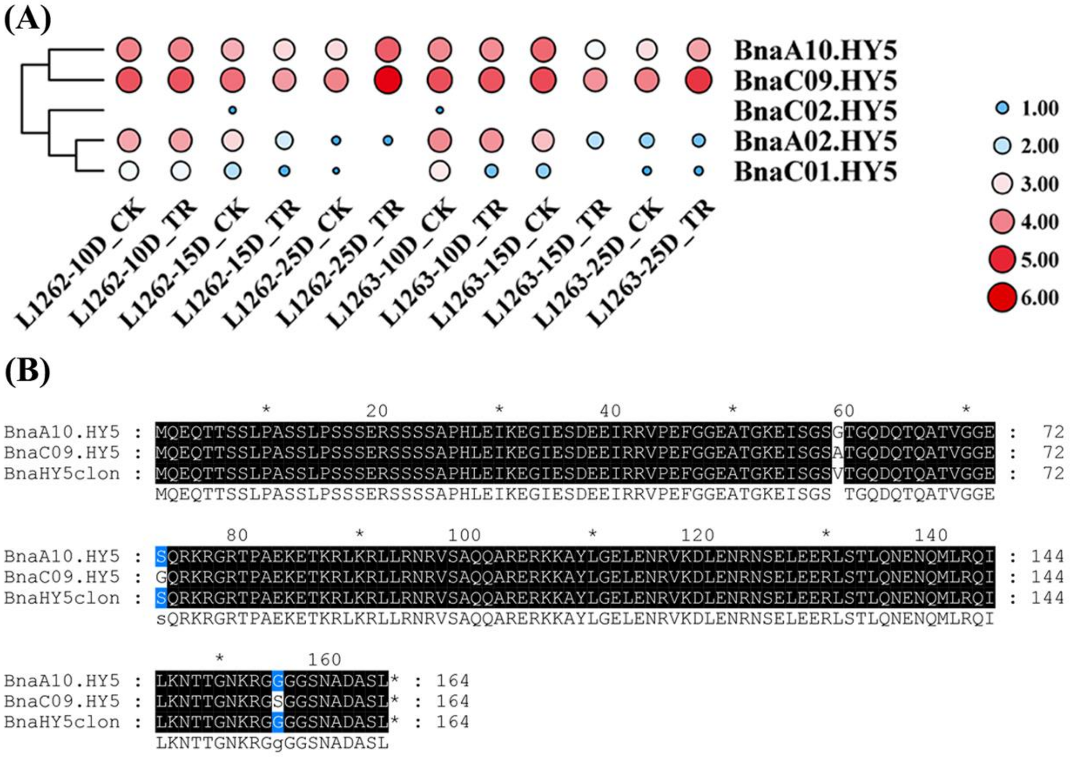

Figure S14. The expression profiles and sequences alignment analysis of BnaHY5 genes. (A) The expression profiles of BnaHY5 genes under shading condition. The expression profiles of each BnaBBX gene are based on log_2-transformed values (FPKM value + 1). FPKM, fragments per kilobase of exon model per million mapped fragments. (B) The sequences alignment of BnaHY5 proteins. BnaHY5 clon represented the amino acid sequence of BnaHY5 obtained through PCR amplification.
